# Supplementary material for: Prediction of differentiation levels in lung adenocarcinoma using peripheral blood inflammatory cytokines and tumor markers
Source: PLoS One. 2026 Jan 8;21(1):e0339414. doi: 10.1371/journal.pone.0339414 (PMC12782445; doi:10.1371/journal.pone.0339414)
Supplement: S1 Fig — (DOCX) [file pone.0339414.s001.docx]

**
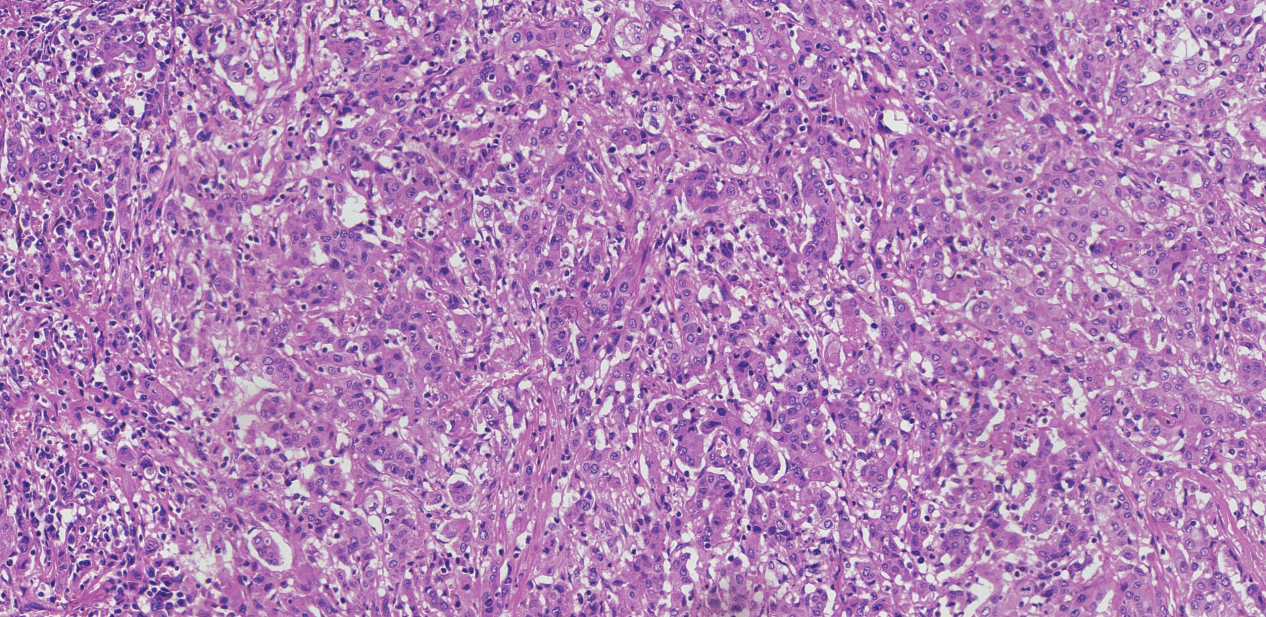
**

**Figure S1** Low differentiated LUAD. Cells exhibit irregular shapes, vary in size, have enlarged and darkly stained nuclei, and feature an increased nucleus-to-cytoplasm ratio, indicating a loss of normal cellular characteristics


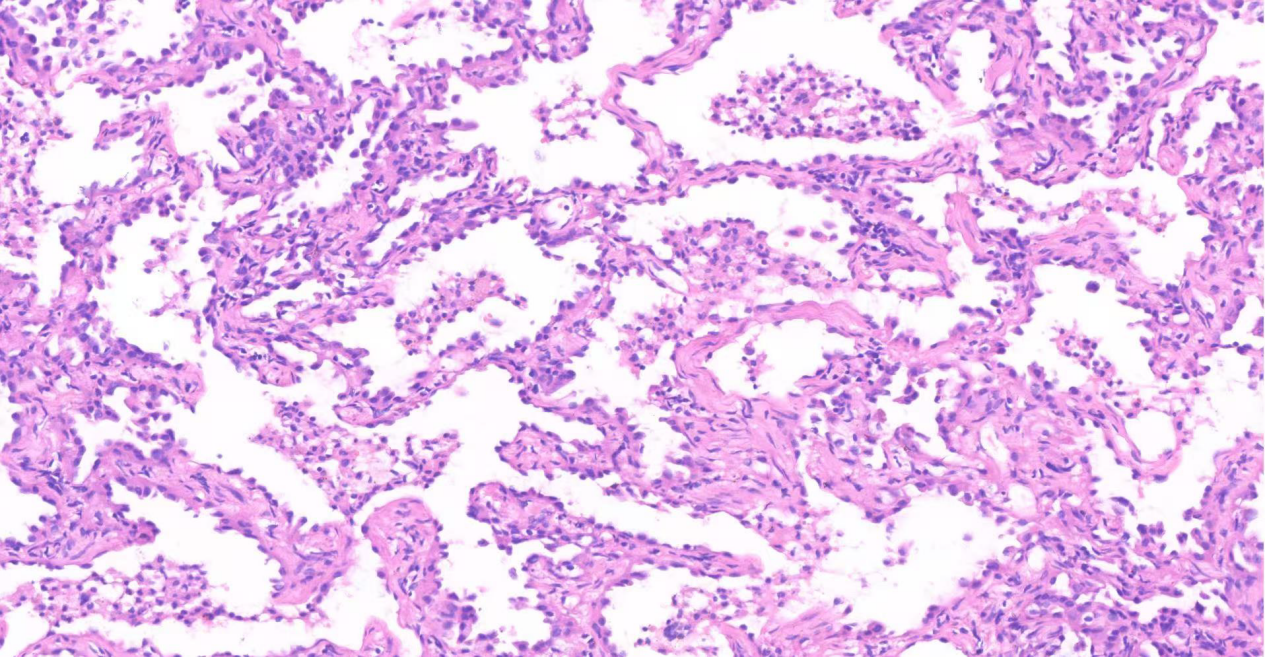


**Figure S2** High differentiated LUAD. Cells exhibit appearance, small nuclei, and a normal nuclear mass ratio. Morphologically, they are very similar to normal lung adenocarcinoma epithelial cells.
