# Supplementary material for: Prediction of differentiation levels in lung adenocarcinoma using peripheral blood inflammatory cytokines and tumor markers
Source: PLoS One. 2026 Jan 8;21(1):e0339414. doi: 10.1371/journal.pone.0339414 (PMC12782445; doi:10.1371/journal.pone.0339414)
Supplement: S1 Table — (DOCX) [file pone.0339414.s002.docx]

| **Table S1** Baseline Characteristics and Laboratory Examinations of Two Groups (the stage 3-4) | | | |
| --- | --- | --- | --- |
| Variables | Low group（n=158） | High group（n=21） | P-value |
| Sex,n(%) |  |  | 0.097 |
| Male | 86 (54.43%) | 16 (76.19%) |  |
| Female | 72 (45.57%) | 5 (23.81%) |  |
| Age(years) | 61.70 ± 11.81 | 63.90 ± 10.20 | 0.369 |
| Height(cm) | 161.14 ± 7.60 | 160.57 ± 7.19 | 0.738 |
| Weight(kg) | 59.49 ± 9.59 | 56.71 ± 7.02 | 0.115 |
| BMI(kg/m2) | 22.91 ± 3.35 | 22.17 ± 3.70 | 0.392 |
| Smoke,n(%) |  |  | 0.391 |
| No | 95 (60.13%) | 10 (47.62%) |  |
| Yes | 63 (39.87%) | 11 (52.38%) |  |
| Drink,n(%) |  |  | 0.195 |
| No | 116 (73.42%) | 12 (57.14%) |  |
| Yes | 42 (26.58%) | 9 (42.86%) |  |
| Diabetes,n(%) |  |  | 0.223 |
| No | 142 (89.87%) | 21 (100.00%) |  |
| Yes | 16 (10.13%) | 0 (0.00%) |  |
| Hypertension,n(%) |  |  | 1.000 |
| No | 118 (74.68%) | 16 (76.19%) |  |
| Yes | 40 (25.32%) | 5 (23.81%) |  |
| CAD,n(%) |  |  | 0.097 |
| No | 151 (95.57%) | 18 (85.71%) |  |
| Yes | 7 (4.43%) | 3 (14.29%) |  |
| Laboratory examination |  |  |  |
| neutrophil | 5.28 ± 3.26 | 4.75 ± 1.36 | 0.186 |
| lymphocyte | 1.40 ± 0.56 | 1.24 ± 0.43 | 0.135 |
| monocyte | 0.51 ± 0.25 | 0.45 ± 0.16 | 0.119 |
| platelet | 242.47 ± 94.77 | 207.29 ± 42.32 | 0.005 |
| lnAISI | 5.94 ± 0.93 | 5.61 ± 0.61 | 0.036 |
| CEA# | 84.00 ± 199.25 | 17.20 ± 39.49 | <0.001 |
| CYFRA21-1# | 7.37 ± 9.64 | 3.73 ± 6.17 | 0.025 |
| Ferritin | 210.95 ± 206.12 | 131.20 ± 110.89 | 0.009 |
| NSE | 16.83 ± 11.80 | 14.64 ± 4.68 | 0.118 |
| SCCA# | 1.14 ± 2.00 | 0.65 ± 0.36 | 0.005 |
| TPA# | 227.38 ± 297.67 | 103.21 ± 126.22 | 0.001 |
| ProGRP# | 40.40 ± 41.31 | 29.82 ± 16.50 | 0.034 |

# Nonnormal data, mean ± standard deviation of non-normal data expressed as mean.

BMI, body mass index; CAD, coronary artery disease; AISI: aggregate index of systemic inflammation; CEA: Carcinoembryonic Antigen; CYFRA21-1: Cytokeratin 19 fragment antigen 21-1; NSE: Neuron Specific Enolase; SCCA: Squamous Cell Carcinoma Antigen; TPA: Tissue Polypeptide Antigen; ProGRP: Progastrin Releasing Peptide.

| **Table S2** Nonnormal Data of Two Groups (the stage 3-4) | | | |
| --- | --- | --- | --- |
| Variables | Low group（n=158） | High group（n=21） | P-value |
| CEA# | 94.89 | 53.24 | <0.001 |
| CYFRA21-1# | 94.44 | 56.62 | 0.002 |
| SCCA# | 93.40 | 64.45 | 0.016 |
| TPA# | 94.40 | 56.90 | 0.002 |
| ProGRP# | 93.34 | 64.86 | 0.018 |

# Nonnormal data, mean ± standard deviation of non-normal data expressed as mean. CEA: Carcinoembryonic Antigen; CYFRA21-1: Cytokeratin 19 fragment antigen 21-1; SCCA: Squamous Cell Carcinoma Antigen; TPA: Tissue Polypeptide Antigen; ProGRP: Progastrin Releasing Peptide.

Table S3 Multicollinearity detection in multiple logistic regression model

| Variable | TOL | VIF |
| --- | --- | --- |
| lnAISI | 0.932 | 1.072 |
| CEA | 0.978 | 1.022 |
| Ferritin | 0.952 | 1.051 |
| ProGRP | 0.997 | 1.003 |

TOL, Tolerance; VIF, Variance inflation factor

A VIF value more than 10 or a TOL less than 0.1 indicate multicollinearity.

AISI: aggregate index of systemic inflammation; CEA: Carcinoembryonic Antigen; ProGRP: Progastrin Releasing Peptide.
