# Supplementary material for: Prediction of differentiation levels in lung adenocarcinoma using peripheral blood inflammatory cytokines and tumor markers
Source: PLoS One. 2026 Jan 8;21(1):e0339414. doi: 10.1371/journal.pone.0339414 (PMC12782445; doi:10.1371/journal.pone.0339414)
Supplement: Attachment — Submitted filename: List of Responses.docx [file pone.0339414.s005.docx]

# List of Responses

Dear Editors and Reviewers:

Thank you for your letter and the reviewers’ comments on our manuscript titled “Prediction of Differentiation Levels in Lung Adenocarcinoma Using Peripheral Blood Inflammatory Cytokines and Tumor Markers” (ID: PONE-D-25-11695). We greatly appreciate the valuable feedback, which has significantly contributed to improving our manuscript and providing essential guidance for our research. We have carefully considered the comments and made the necessary revisions, which we hope will meet your approval. The revised sections are highlighted in red in the manuscript. Below are the main corrections and our responses to the reviewers’ comments:

**Reviewer 1:**

**Comment1: Please decrypt acronyms in abstract.**

Response：Thank you for your valuable feedback.We have introduced the acronyms in the abstract and highlighted them in red.Thank you once again. Your valuable insights have significantly contributed to refining and uplifting the quality of our paper.

**Comment2:**

**All tables presentation need to be improved. Table 2, 3 at the moment do not have borders and it is difficult to read. Please consider organizing and presenting better.**

Response：

Thank you for pointing this out.In the new manuscript, readers can have a better understanding of the tables in this study. We have added corresponding borders to the tables and beautified the table format.Your insights have been immensely valuable in shaping and enhancing the overall quality of our paper.

**Comment3:**

**Table 2: CYFRA21/SCCA TPA abbreviations? P25/P50 meaning?**

Response:

Thank you for the question.P25 and P50, as key percentile statistics, play an irreplaceable role in the research. P25 provides a benchmark for the low-value range, risk threshold setting and outlier identification by marking the lower quartiles of the data distribution. While P50, as the median, accurately reflects the central trend of the data, especially being more representative in the skewed distribution. The combined use of the two can comprehensively depict the distribution characteristics of the data (such as lower limits, centers and skewness), providing an important basis for risk assessment, inter-group comparison and outlier detection. This study introduces these two indicators, aiming to reveal the inherent laws of the data more accurately and lay a solid foundation for subsequent analysis.Your insights have been immensely valuable in shaping and enhancing the overall quality of our paper.

**Comment4:**

**Table 3: High group N=44 and Low group N=159. This difference is nearly 4-folds. I am not exactly certain how the statistics problem will be solved by this difference. The statistics is skewed and compelling the findings become dubious to interpret. At the moment Figure 3,4 is not helping either to understand weightage.**

Response：

Thank you for your valuable feedback.The reasons for this imbalance may be multifaceted. Firstly, from the patients' perspective, high-differentiation patients may perceive their condition as relatively mild with unobvious symptoms, leading to a less urgent need for medical attention. Additionally, economic factors could play a significant role. Some patients, particularly those with limited financial resources, may hesitate to seek medical care or delay their visits due to concerns about treatment costs.Furthermore, psychological factors should not be overlooked. High-differentiation patients may hold a more optimistic view of their disease prognosis and, consequently, lack the motivation to actively seek medical help.

Meanwhile In our study, we utilized a binary logistic regression model for analysis. When the sample size exceeds 200, the bias in parameter estimation can be considered negligible and therefore does not need to be taken into account^[1]^.

In addition due to the nature of this study as a real-world investigation, the data was collected from a single center. Within the limited timeframe, the number of cases of undifferentiated or moderately differentiated adenocarcinoma was too restricted to allow for robust statistical analysis. Focusing on these two groups can enable a more precise research focus, enhance the accuracy of the study, and generate data that is more relevant to clinical decision-making.Thank you once again. Your valuable insights have significantly contributed to refining and uplifting the quality of our paper.

Reference: 1.Chen, B. (2012). "Medical multivariate analysis design sample size estimation—A comprehensive estimation method for multivariate analysis design sample size." Shanghai Medical Journal (Electronic Edition), 1.04, 58-60.(In chinese)

**Comment5:**

**None of the figures have legends. Figure 2-4 are incomprehensible at the moment.**

Response：

Thank you for pointing this out.At the end of the manuscript, we have added legends to Figures 2-6 to facilitate readers' better reading and understanding.Your insights have been immensely valuable in shaping and enhancing the overall quality of our paper.

**Reviewer 2:**

**Comment1:**

**However, the authors should take care to define all abbreviations used in the article as this was not done.**

Response:

Thank you for your valuable comment.In the new manuscript, we reinterpreted all the abbreviations.Your feedback has been incredibly beneficial in enhancing the quality of our paper.

**Comment2:**

**Whilst the authors have a statistics analysis method, more detail can be included here considering that the entire predictive model is based on this methodology. For instance, the authors could elaborate on the methodology for generating the decision curve Figure 6. The same applies to the AUC of the nomogram model (Figure 4) and the calibration curve (Figure 5).**

Response:

Thank you for your valuable comment.The methods for generating relevant curves and Nomogram models are mentioned in the statistical methods of this paper. Meanwhile, at the end of the new manuscript, we further explained the pictures in the study.Thank you again for your valuable feedback and for helping us to improve the quality and accuracy of our study.

**Comment3:**

Response:

Thank you for your valuable comment.To systematically clarify the differences between Low Differentiated and High Differentiated lung adenocarcinoma, the following methods were adopted in this study:Sample selection and grouping criteria.

Sample source: Pathologically confirmed cases of lung adenocarcinoma (n=203) collected from the Second Affiliated Hospital of Chongqing Medical University, with a time span of [June 1, 2022 to July 30, 2024].

Grouping basis: According to the 2021 classification criteria for lung tumors of the World Health Organization (WHO), two senior pathologists independently reviewed the pathological sections. Based on characteristics such as the proportion of acini/papilla/micropapilla/solid type and nuclear atypia, the samples were divided into the LD group (Low Differentiated) and the HD group (High differentiated).Thank you for your understanding and valuable feedback.Due to the limitations of width and height, we have improved the quality of the pictures as much as possible as required by the magazine. Currently, there are clearer PDF versions of pictures 2 to 6, which have now been uploaded to the supplementary materials section.

**Comment4:**

**All figures are poor in quality - the resolution of each figure needs to be improved. Furthermore, figures have not been presented with figure legends.**

Response:

Due to the limitations of width and height, we have improved the quality of the pictures as much as possible as required by the magazine. Currently, there are clearer PDF versions of pictures 2 to 6, which have now been uploaded to the supplementary materials section.At the end of the manuscript, we have added legends to Figures 2-6 to facilitate readers' better reading and understanding.Thank you again for your valuable feedback and for helping us to improve the quality and accuracy of our study.

**Comment5:**

**Furthermore, an introductory sentence or two needs to be added to the abstract.**

Response:

Thank you for your comment.We have added appropriate introduction sentences in the abstract to facilitate readers' better reading and understanding.Thank you once again for your valuable feedback.
