# Supplementary material for: Prediction of differentiation levels in lung adenocarcinoma using peripheral blood inflammatory cytokines and tumor markers
Source: PLoS One. 2026 Jan 8;21(1):e0339414. doi: 10.1371/journal.pone.0339414 (PMC12782445; doi:10.1371/journal.pone.0339414)
Supplement: Attachment — Submitted filename: response.docx [file pone.0339414.s006.docx]

**List of Responses**

Dear Editor and Reviewers:

Thank you for your letter and for the reviewers’ comments concerning our manuscript entitled “Prediction of Differentiation Levels in Lung Adenocarcinoma Using Peripheral Blood Inflammatory Cytokines and Tumor Markers” (**ID: PONE-D-25-11695R1**). Those comments are all valuable and very helpful for revising and improving our paper, as well as the important guiding significance to our researches. We have studied comments carefully and have made correction which we hope meet with approval. Revised portion are marked in red in the paper. The main corrections in the paper and the responds to the reviewer’s comments are as flowing:

Responds to the comments of editor and reviewers:

**Reviewer #1:**

**1.Comment:** *If the authors have adequately addressed your comments raised in a previous round of review and you feel that this manuscript is now acceptable for publication, you may indicate that here to bypass the “Comments to the Author” section, enter your conflict of interest statement in the “Confidential to Editor” section, and submit your "Accept" recommendation.* **All comments have been addressed**

**Response: Thank you very much for your recognition of this manuscript.**

**2.Comment:** *Is the manuscript technically sound, and do the data support the conclusions?The manuscript must describe a technically sound piece of scientific research with data that supports the conclusions. Experiments must have been conducted rigorously, with appropriate controls, replication, and sample sizes. The conclusions must be drawn appropriately based on the data presented.* **Yes**

**Response: Thank you very much for your recognition of this manuscript.**

**3. Comment:** *Has the statistical analysis been performed appropriately and rigorously?* **Yes**

**Response: Thank you very much for your recognition of this manuscript.**

**4.Comment:** *Have the authors made all data underlying the findings in their manuscript fully available? The PLOS Data policy requires authors to make all data underlying the findings described in their manuscript fully available without restriction, with rare exception (please refer to the Data Availability Statement in the manuscript PDF file). The data should be provided as part of the manuscript or its supporting information, or deposited to a public repository. For example, in addition to summary statistics, the data points behind means, medians and variance measures should be available. If there are restrictions on publicly sharing data—e.g. participant privacy or use of data from a third party—those must be specified.* **Yes**

**Response: Thank you very much for your recognition of this manuscript.**

**5.Comment:** *Is the manuscript presented in an intelligible fashion and written in standard English?PLOS ONE does not copyedit accepted manuscripts, so the language in submitted articles must be clear, correct, and unambiguous. Any typographical or grammatical errors should be corrected at revision, so please note any specific errors here.* **Yes**

**Response: Thank you very much for your recognition of this manuscript.**

**6.Comment:** *Review Comments to the Author. Please use the space provided to explain your answers to the questions above. You may also include additional comments for the author, including concerns about dual publication, research ethics, or publication ethics. (Please upload your review as an attachment if it exceeds 20,000 characters).*

Reviewer #1: The article titled "Prediction of Differentiation Levels in Lung Adenocarcinoma Using Peripheral Blood Inflammatory Cytokines and Tumor Markers" certainly is novel and the connecting findings are clinically relevant with abundance of upcoming literature in this area. I have read the manuscript and the response to previous reviewer's comments. I do believe addition of the ROC/AUC, decision curve analysis certainly improved from the previous version. I have a few comments that I urge the authors to consider for another revision.

1) The sensitivity analysis and potential of overfitting risks must be thoroughly explained in the discussion. Overall the limitations section need to be more thorough for this study. If possible please expand on external validation studies, potential geographic area bias. A few future directions on multi-center studies and tumor microenvironment profiling/single cell studies etc. will address these limitations.

**Response: Thank you very much for your valuable and insightful comments. We have substantively revised and expanded the Discussion section of our manuscript based on your suggestions. To assess the robustness of the model, we performed Bootstrap resampling to evaluate model performance. The results showed an absolute error of 0.012 between the simulated curve and the actual curve, indicating a high degree of similarity and consistency in the trends of the two curves. Furthermore, the model achieved an AUC of 0.795 (95% CI: 0.726–0.864), which strengthens our confidence in its reliability. Additionally, to mitigate the risk of overfitting, we have supplemented the analysis with multicollinearity test results (Supplementary Table 3), which demonstrated no collinearity among the variables. However, limitations must be acknowledged. First, although multicollinearity tests were employed during model construction to reduce the risk of overfitting, this risk remains in studies with limited sample sizes. Second, the retrospective data used in this study were solely sourced from the Second Affiliated Hospital of Chongqing Medical University, which may introduce potential geographic and population biases. We acknowledged that the model developed from this single center dataset may have limited generalizability, and its performance could decline when applied to populations from other regions, with different ethnic backgrounds, or under varying healthcare settings. Third, we recognized that a truly robust model requires validation with data from other independent centers, but the step had not yet been accomplished in this study. To directly address the limitations identified in this study, we propose several key directions for future research. First, multi center collaborations should be initiated to conduct rigorous external validation of our current model using independent datasets from various institutions. This is an essential step for confirming the model's clinical utility and generalizability. Second, large scale prospective cohort studies are warranted to elucidate the temporal relationship between AISI and the incidence and progression of lung adenocarcinoma. Third, integrating more precise diagnostic tools for lung adenocarcinoma differentiation could enhance the accuracy and reliability of research findings. Furthermore, developing predictive models that incorporate AISI with other biomarkers would help improve prognostic accuracy. Finally, fundamental experimental research focusing on areas such as tumor microenvironment analysis and single-cell studies should be pursued to progressively address the current limitations of the research.**

2) For the CEA please explain how borderline p values were handled to avoid misinterpretations, Page 14 "The original p-value for CEA was 0.0497.....".

**Response: We thank the reviewer for this insightful and important comment regarding the interpretation of borderline p-values of CEA. We fully agree that p-values close to the conventional alpha level of 0.05 require careful handling to prevent overinterpretation due to random chance. The p-value for CEA was 0.0497, indicating statistical significance at the 0.05 level. Therefore, we have chosen to retain the value of 0.0497, rather than 0.05 or <0.05. This approach effectively prevents potential misinterpretation while clearly informing readers of the statistical rationale for including CEA in the final model.**

3) I think funding for this study is mentioned twice. Please check acknowledgements and funding sections.

**Response: Thank you for your valuable feedback. We have revised in the revised manuscript. Acknowledgments: We thank the Second Affiliated Hospital of Chongqing Medical University for their support. Funding: This work was supported by Application of single-cell biomolecular analysis in the pathogenesis of lung cancer(cstc2022yejh-bgzxm0051).**

4) Figure resolutions need to enhance.

**Response: Thank you for your valuable feedback. The figures have been remade and saved in TIFF format (Figure1 - Figure6).**

5) The abstract should report confidence intervals(95%)for AUC And clarifying the relevance of ProGRP (for the revised manuscript) for the context of LUAD.

**Response: Thank you for your valuable feedback. We have revised in the revised manuscript. AISI (OR = 1.64, 95% CI = 1.08-2.58, p = 0.024), CEA (OR = 1.02, 95% CI = 1.00-1.04, p = 0.0497), ferritin (OR = 1.01, 95% CI = 1.00-1.01, p = 0.010), and Progastrin Releasing Peptide (ProGRP) (OR = 1.03, 95% CI = 1.00-1.07, p = 0.047) were risk factors of low differentiation LUAD. The model achieved an Area Under Curve(AUC) of 0.795 (95%CI: 0.726-0.864).**

6) Waiving of informed consent must be clearly declared in methods. Proofread throughout for textual errors.

**Response: Thank you for your valuable feedback. We have revised in the revised manuscript. All personal identifiers were removed prior to researcher access, and this retrospective study was conducted in accordance with the ethical principles of the Declaration of Helsinki and was approved by the Second Affiliated Hospital of Chongqing Medical University Ethics Committee (No.85, 2024), with a waiver of informed consent obtained. Additionally, we have thoroughly proofread the manuscript to correct any spelling errors.**

7) Please consider providing more detailed how decision curve analysis and calibration curves were generated in R/bootstrap approach.

**Response: Thank you for your valuable feedback. We have revised in the revised manuscript. The calibration curve was plotted using the Bootstrap method with 1,000 repetitions. The dashed diagonal line represents the ideal line, which signifies a perfect prediction. The solid line indicates the actual prediction performance of the model. The closer these two lines are to each other, the better the model's predictive accuracy. The results are presented in Figure 5. The absolute error between the simulated curve and the actual curve is 0.012, demonstrating that the two curves follow a highly similar trend and exhibit strong consistency. The decision curve for the established nomogram was constructed. The clinical value of this model increases as its curve moves away from the "treat all" or "treat none" curves. The threshold at which patients benefit from this model ranges from 30% to 95%. Furthermore, the decision curve analysis depicted in Figure 6 confirms the clinical utility of the model, demonstrating a favorable net benefit rate.**

**7.Comment:** *PLOS authors have the option to publish the peer review history of their article (what does this mean?). If published, this will include your full peer review and any attached files. If you choose “no”, your identity will remain anonymous but your review may still be made public. Do you want your identity to be public for this peer review? For information about this choice, including consent withdrawal, please see our Privacy Policy.* ***Yes: Joydeep Chakraborty***

**Response: Thank you very much for your recognition of this manuscript. Your valuable comments are of great importance for the improvement of this manuscript.**

**Reviewer #2:**

The authors set out to investigate the predictive value of a combination of peripheral blood inflammatory markers and tumour markers to determine the differentiation levels of lung adenocarcinoma. The peripheral blood biomarkers included neutrophils, monocytes, platelets, and age. The hypothesis stated that certain marker combinations could more accurately reflect the biological characteristics of lung adenocarcinoma. The authors developed a predictive model with an AUC of 0.795, which led them to claim that the peripheral blood biomarkers (including neutrophils, monocytes, platelets, AISI), age, and tumor biomarkers (CEA and ProGRP) are associated with pathological differentiation in lung adenocarcinoma. The claims are significant with respect to advances in biomarker-driven oncology. The model is non-invasive and cost-effective, and may serve as potential predictors for the differentiation of lung adenocarcinoma and may guide personalized treatment planning.

The study presents the results of original research, and has not been published elsewhere. The data presented in the manuscript support the conclusions drawn.

The authors have done well to meet the applicable standards for the ethics requirements of the study.

**Response: Thank you very much for your recognition of this manuscript.**

The authors have placed the study in context with literature. However, the authors should take care to define all abbreviations used in the article – as this was not done. The data and analyses support the authors claims.

**Response: Thank you for your valuable feedback. We have revised in the revised manuscript. LUAD: lung adenocarcinoma ; BMI, body mass index; CAD, coronary artery disease; AISI: aggregate index of systemic inflammation; CEA: Carcinoembryonic Antigen; CYFRA21-1: Cytokeratin 19 fragment antigen 21-1; NSE: Neuron Specific Enolase; SCCA: Squamous Cell Carcinoma Antigen; TPA: Tissue Polypeptide Antigen; ProGRP: Progastrin Releasing Peptide.**

*Whilst the authors have a statistics analysis method, more detail can be included here – considering that the entire predictive model is based on this methodology. For instance, the authors could elaborate on the methodology for generating the decision curve – Figure 6. The same applies to the AUC of the nomogram model (Figure 4) and the calibration curve (Figure 5). Furthermore, the heading “Definition of low and high differentiation” explains the differences between low- and high-differentiated lung adenocarcinoma but includes no methodology. It would have been more thorough for the authors to show cell images representing the differences*

*taken from patient samples, and the authors could consider including this in the manuscript.*

**Response: Thank you for your valuable feedback. We have revised in the revised manuscript. The calibration curve was plotted using the Bootstrap method with 1,000 repetitions. The dashed diagonal line represents the ideal line, which signifies a perfect prediction. The solid line indicates the actual prediction performance of the model. The closer these two lines are to each other, the better the model's predictive accuracy. The results are presented in Figure 5. The absolute error between the simulated curve and the actual curve is 0.012, demonstrating that the two curves follow a highly similar trend and exhibit strong consistency. The decision curve for the established nomogram was constructed. The clinical value of this model increases as its curve moves away from the "treat all" or "treat none" curves. The threshold at which patients benefit from this model ranges from 30% to 95%. Furthermore, the decision curve analysis depicted in Figure 6 confirms the clinical utility of the model, demonstrating a favorable net benefit rate. Low differentiated LUAD refers to cancer cells with minimal differentiation, showing marked deviations from the epithelial cells of normal lung glands. These cells exhibit irregular shapes, vary in size, have enlarged and darkly stained nuclei, and feature an increased nucleus-to-cytoplasm ratio, indicating a loss of normal cellular characteristics (Figure SIa). High differentiated LUAD consists of cancer cells that are highly differentiated, closely resembling normal lung gland epithelial cells in morphology. These cells appear regular, with smaller nuclei and a normal nucleus-to-cytoplasm ratio(Figure SIb) .**

All figures are poor in quality - the resolution of each figure needs to be improved. Furthermore, figures have not been presented with figure legends.

**Response: Thank you for your valuable feedback. We have revised in the revised manuscript. The figures have been remade and saved in TIFF format. Figure legends were shown in revised manuscript.**

Whilst the manuscript is written clearly, numerous editorial errors need to be corrected. Furthermore, the authors should define all abbreviations so that the information is accessible to non-specialists. Furthermore, an introductory sentence or two must be added to the abstract.

**Response: Thank you for your valuable feedback. We have revised in the revised manuscript. We further revised the language of the manuscript to ensure readability. Additionally, we have included a glossary of abbreviations. We have added an introductory sentence in the abstract. Lung Adenocarcinoma (LUAD) has highly aggressive and lethal, and its degree of differentiation significantly influences prognosis and treatment strategies, yet accurate prediction remains challenging.**

We tried our best to improve the manuscript and made some changes in the manuscript. These changes will not influence the content and framework of the paper. And here we did not list the changes but marked in red in revised paper.

We appreciate for Editors/Reviewers’ warm work earnestly, and hope that the correction will meet with approval.

Once again, thank you very much for your comments and suggestions.
