# Supplementary material for: Prediction of differentiation levels in lung adenocarcinoma using peripheral blood inflammatory cytokines and tumor markers
Source: PLoS One. 2026 Jan 8;21(1):e0339414. doi: 10.1371/journal.pone.0339414 (PMC12782445; doi:10.1371/journal.pone.0339414)
Supplement: Attachment — Submitted filename: Reviewer comments.pdf [file pone.0339414.s003.pdf]

Reviewer Comments

The authors set out to investigate the predictive value of a combination of peripheral blood inflammatory markers and tumour markers to determine the differentiation levels of lung adenocarcinoma. The peripheral blood biomarkers included neutrophils, monocytes, platelets, and age. The hypothesis stated that certain marker combinations could more accurately reflect the biological characteristics of lung adenocarcinoma. The authors developed a predictive model with an AUC of 0.795, which led them to claim that the peripheral blood biomarkers (including neutrophils, monocytes, platelets, AISI), age, and tumor biomarkers (CEA and ProGRP) are associated with pathological differentiation in lung adenocarcinoma. The claims are significant with respect to advances in biomarker-driven oncology. The model is non-invasive and cost-effective, and may serve as potential predictors for the differentiation of lung adenocarcinoma and may guide personalized treatment planning.

The study presents the results of original research, and has not been published elsewhere. The data presented in the manuscript support the conclusions drawn.

The authors have done well to meet the applicable standards for the ethics requirements of the study.

The authors have placed the study in context with literature. However, the authors should take care to define all abbreviations used in the article – as this was not done.

The data and analyses support the authors claims.

Whilst the authors have a statistics analysis method, more detail can be included here – considering that the entire predictive model is based on this methodology. For instance, the authors could elaborate on the methodology for generating the decision curve – Figure 6. The same applies to the AUC of the nomogram model (Figure 4) and the calibration curve (Figure 5). Furthermore, the heading “Definition of low and high differentiation” explains the differences between low- and high-differentiated lung adenocarcinoma but includes no methodology. It would have been more thorough for the authors to show cell images representing the differences taken from patient samples, and the authors could consider including this in the manuscript.

All figures are poor in quality - the resolution of each figure needs to be improved. Furthermore, figures have not been presented with figure legends.

Whilst the manuscript is written clearly, numerous editorial errors need to be corrected. Furthermore, the authors should define all abbreviations so that the information is accessible to non-specialists. Furthermore, an introductory sentence or two must be added to the abstract.

Recommendation:

Major revision required.

The authors should certainly be encouraged to resubmit a revised version as this article could further the current understanding of predictors for the differentiation of lung adenocarcinoma.
